# Partial nephrectomy using radiofrequency incremental bipolar generator with multi electrode probe: experimental study in bench pig kidneys

**DOI:** 10.1186/1471-2490-14-7

**Published:** 2014-01-10

**Authors:** Piero Rossi, Pierluigi Bove, Mauro Montuori, Adriano De Majo, Edoardo Ricciardi, Maurizio Mattei, Roberta Bernardini, Luigino Calzetta, Paolo Mauti, Lorenzo Intini, Valentino Quattrini, Carlo Chiaramonte, Giuseppe Vespasiani

**Affiliations:** 1 Department of General Surgery, University Hospital of Tor Vergata, Rome, Italy; 2 Department of Urology, University Hospital of Tor Vergata, Rome, Italy; 3 Department of Biology STA, University Hospital of Tor Vergata, Rome, Italy; 4 Laboratory of Systems Approaches and Non-Communicable Diseases, San Raffaele Pisana Hospital, IRCCS, Rome, Italy; 5 L.E.D. S.p.a., Aprilia, Latina, Italy; 6 Department of Statistics, University Hospital of Tor Vergata, Rome, Italy

**Keywords:** Nephron sparing surgery, Partial nephrectomy, Bipolar radiofrequency, Multi-electrode probe, LaparoNewPro

## Abstract

**Background:**

The aim of this research project was the realization of an incremental bipolar radiofrequency generator with inline 4-electrode probe for partial renal resection without clamping of the vessels.

**Methods:**

The experimentation was carried out across two phases: the preliminary realization of a specific generator and an inline multielectrode probe for open surgery (Phase 1); system testing on 27 bench kidneys for a total of 47 partial resection (Phase 2).

The parameters evaluated were: power level, generator automatisms, parenchymal coagulation times, needle caliber, thickness of the coagulated tissue “slice”, charring, ergonomy, feasibility of the application of “bolster” stitches.

**Results:**

The analysis of the results referred to the homogeneity and thickness of coagulation, energy supply times with reference to the power level and caliber of the needles. The optimal results were obtained by using needles of 1.5 mm caliber at power level 5, and with coagulation times of 54 seconds for the first insertion and 30 seconds for the second.

**Conclusions:**

The experimentation demonstrated that the apparatus, consisting of a generator named “*LaparoNewPro*” and fitted with a dedicated probe for open surgery, is able to carry out a coagulation of the line of resection of the renal parenchyma in a homogeneous manner, in short times, without tissue charring, and with the possibility of stitching both on coagulated tissue and the caliceal system.

The generator automatism based on the flow of the current supplied by each electrode is reliable, and the cessation of energy supply coincides with optimal coagulation.

## Background

The diffusion of screening programs and the use of modern techniques of imaging [1] have led to an earlier diagnosis of renal neoplasms [2,3].

Radical nephrectomy was always considered the therapeutic gold standard of renal tumours up to the early 90’s, when both the minimal invasive and partial renal resection approaches, as Nephron Sparing Surgery (NSS), had became established.

Nephron Sparing Surgery in localized renal tumours demonstrated an oncological outcome comparable with radical nephrectomy [4], with better long-term preservation of the renal function and increased overall survival [5,6].

NSS is the gold standard in tumours of ≤ 4 centimetres (pT1a) [7]; and in selected cases in the T1b (4 – 7 cm) forms [8].

### Control of bleeding is the principal difficulty in NSS

Many devices and haemostatic agents [9] are available in order to improve haemostasis.

For hepatic surgery, the Radio-Frequency assisted Liver resection [10] technique (RFA-LR) has been developed, consisting of the coagulation of a slice of parenchyma preliminary to transection. In this connection, we planned and realized a generator and a dedicated multi-needle inline probe [11,12].

Its main principle in hepatic surgery is that, the “comb” arrangement of the electrode-needles permits the realization of coagulation of “slice” of the parenchyma around the neoplastic nodules, or along anatomic planes with closure of the blood and biliary vessels, without the Pringle maneuver and with little or no blood loss. The coagulated tissue can be transected by a scalpel.

That principle can be applied in theory to all parenchymas.

In order to perform a “blood-less” partial renal resection without clamping, we have developed, in collaboration with the LED S.p.A. company (Aprilia, Italy), a research project for the realization of an original and specific generator together with a multi-electrode probe for both open and laparoscopic approaches.

The project is organized in various phases, that have included the joint work of researchers of LED S.p.A., the Department of Mechanical Engineering, for the designing of the laparoscopic probe, of researchers from the Departments of Surgery and Urology, and the Animal Technology Station (S.T.A.), of the University of Tor Vergata, Rome respectively.

In this manuscript, we report the results of the first two phases of experimentation related to the realization of the specific generator and the inline multi-electrode probe for open surgery, tested on 27 bench kidneys for a total of 47 partial resections.

## Methods

The experimentation has been carried out in two phases. During the first, the dedicated generator and the four-needle inline probe were developed and set up. In the second phases the device was tested on 27 bench kidneys for a total of 47 polar resections.

### Phase 1

#### ***Technical details of the generator***

The system consists of an incremental and bipolar voltage square wave generator, supplying a frequency of 350 kHz and of six output channels, and each channel has a greater voltage than preceding.

Before the beginning of treatment, the user can select a power level between 1 and 9.

Higher power values involve less treatment duration, and thus a thinner coagulation route. In practice, the thickness of the coagulation slice is between 6 mm and 8 mm.

The delivered power is inversely proportional to the impedance. The generator is able to maintain the power density proportional to the tissue volume.

During the treatment, current flow change according to the impedance variation (as a result of tissue drying) decreasing the probability of carbonization.

The current in the comb is outgoing from the first electrode and incoming in the last electrode, limiting the problem of a rapid exsiccation and adherence of the tissue on the needles, due to the high current density in proximity of the electrode.

The system, checking the currents in the electrodes, is able to finish the treatment automatically when the current has decreased below the 30% of its maximum value during the treatment.

### Phase 2

27 experimental tests were carried out on pig kidneys for a total of 47 polar resections.

The four-needle probe was inserted at the renal pole and coagulation was started.

**Figure 1:**
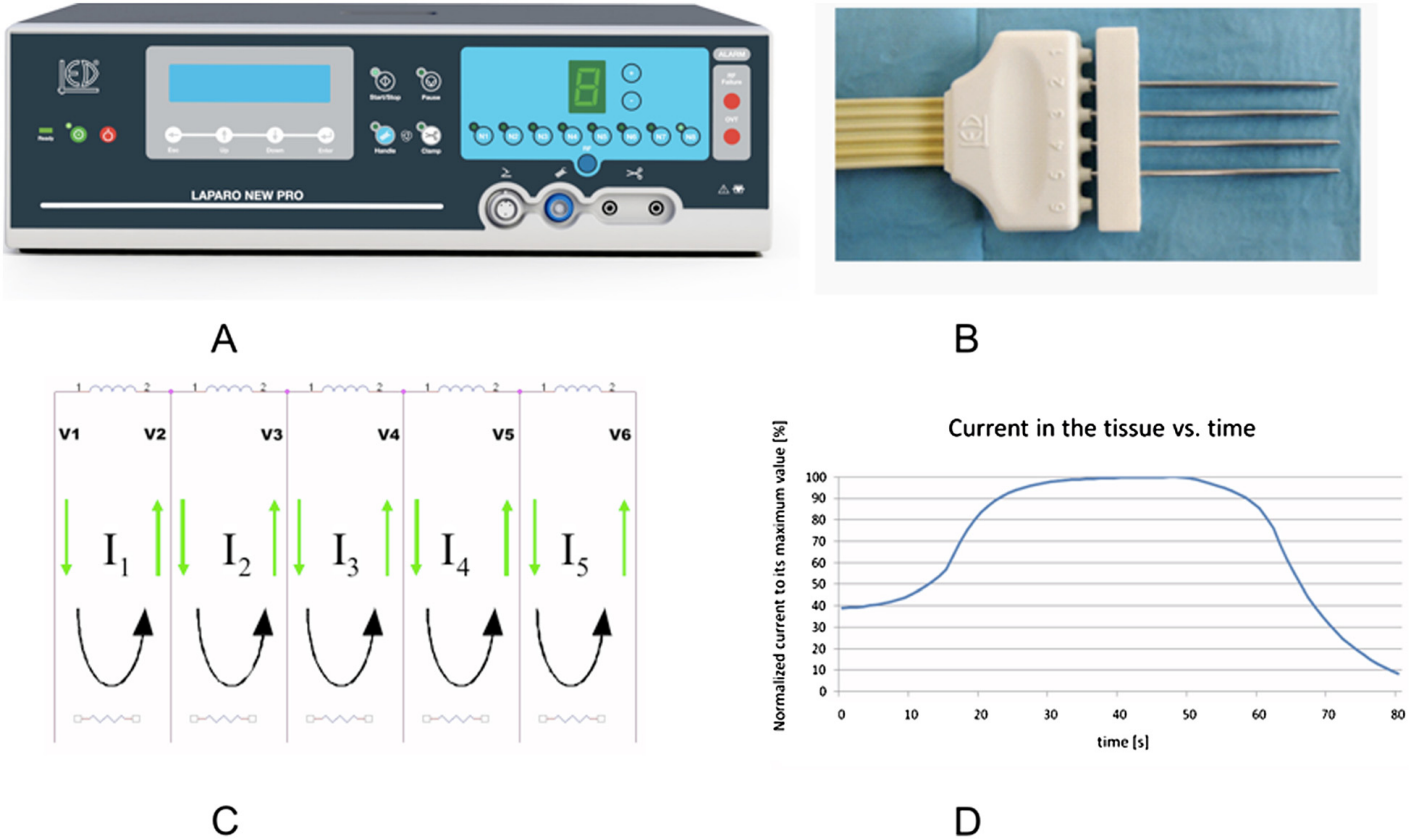
A) Generator LaparoNewPro; B) Probe; C) Current flow; D) Trend of the current into the tissue as a function of time.

Cessation of energy supply by the generator took place automatically and adequate parenchymal coagulation was obtained (Figure 1).

At the end of the coagulation procedure, the time from the start to the automatic stop of the generator was obtained for each insertion.

The maneuver was repeated along the line, inserting the probe until completion of the first line.

**Figure 2:**
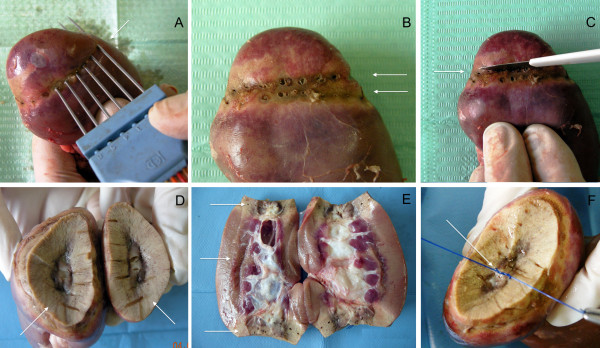
**Steps of the partial resection with the use of Laparonewpro.**  First Line of Coagulation **(A)**; Double Coagularion Line **(B)**; Transection with scalpel **(C)**; Edges **(D)**; Thikness of Coagulation slices **(E)**; Stiches on Caliceal System **(F)**.

Successively, a second, distal, line of insertion of the probe was carried out at about 5 mm from the first (Figure 2).

At the end of the second line, a caliper was used to measure the slice of coagulated tissue.

Four-needle conical-pointed probes were used.

The needles used were of 1.5 mm and 1 mm caliber; the power levels tested were levels 1, 3, 5, 6, 7 and 9.

With the 1 mm needle, we tested power level 5 in 6 polar resections, level 7 in 4 resections and level 9 in 5 polar resections.

With the 1.5 mm needle, tests were power level 1 in 2 cases, level 3 in 2 resections, level 5 in 9 resections, level 6 in 2, level 7 in 4, and level 9 in 13 polar resections.

Following coagulation, the parenchyma was sectioned with a scalpel along the distal line of insertion.

In 10 polar resections, we verified the possibility of applying stitches (Monocryl 2/0) on the coagulated renal parenchyma and on the exposed caliceal system following resection.

The parameters evaluated were: needle caliber, coagulation times, power level, thickness of the “slice” of coagulated tissue, eventual charring, ease of handling during the insertion and removal phases of the probe, feasibility of the application of stitches on the parenchyma and the caliceal system, and generator automatisms.

## Results

### 47 polar resections were carried out on 27 pig kidneys

The coagulation slices following the double coagulation line were homogeneous, with a thickness of about 1 cm.

In experimentation with needles of 1 mm, average coagulation times for first-line insertion were between 33 seconds (Level 9 power) and 52 seconds (Level 5 power) respectively, and, for second-line insertions, between 19 seconds (Level 9 Power) and 29 seconds (Level 5 Power) respectively.

With needles of 1.5 mm, first-line insertion times varied between 32 seconds (Level 9 Power) and 109 seconds (Level 1 Power), while those of the second line were between 15 seconds (Level 9) and 66 seconds (Level 1).

The experiments with 1.5 mm needles and power levels 1 and 3 were not analyzed statistically as it was obvious that such low power levels gave too long coagulation times and produced excessive thickness (approx. 1.5 cm), and power level 6 was not analyzed statistically due to the low number of tests.

The experimental phase on bench kidneys allowed us to specify that the optimal caliber of the electrode-needles is 1.5 mm, because this caliber offer an adeguate rigidity of electrode-needles.

The conical point allows simple insertion in the parenchyma and reduces the risk of injury to the hand of the surgeon and the surrounding organs as compared with the pyramidal point.

**Figure 3:**
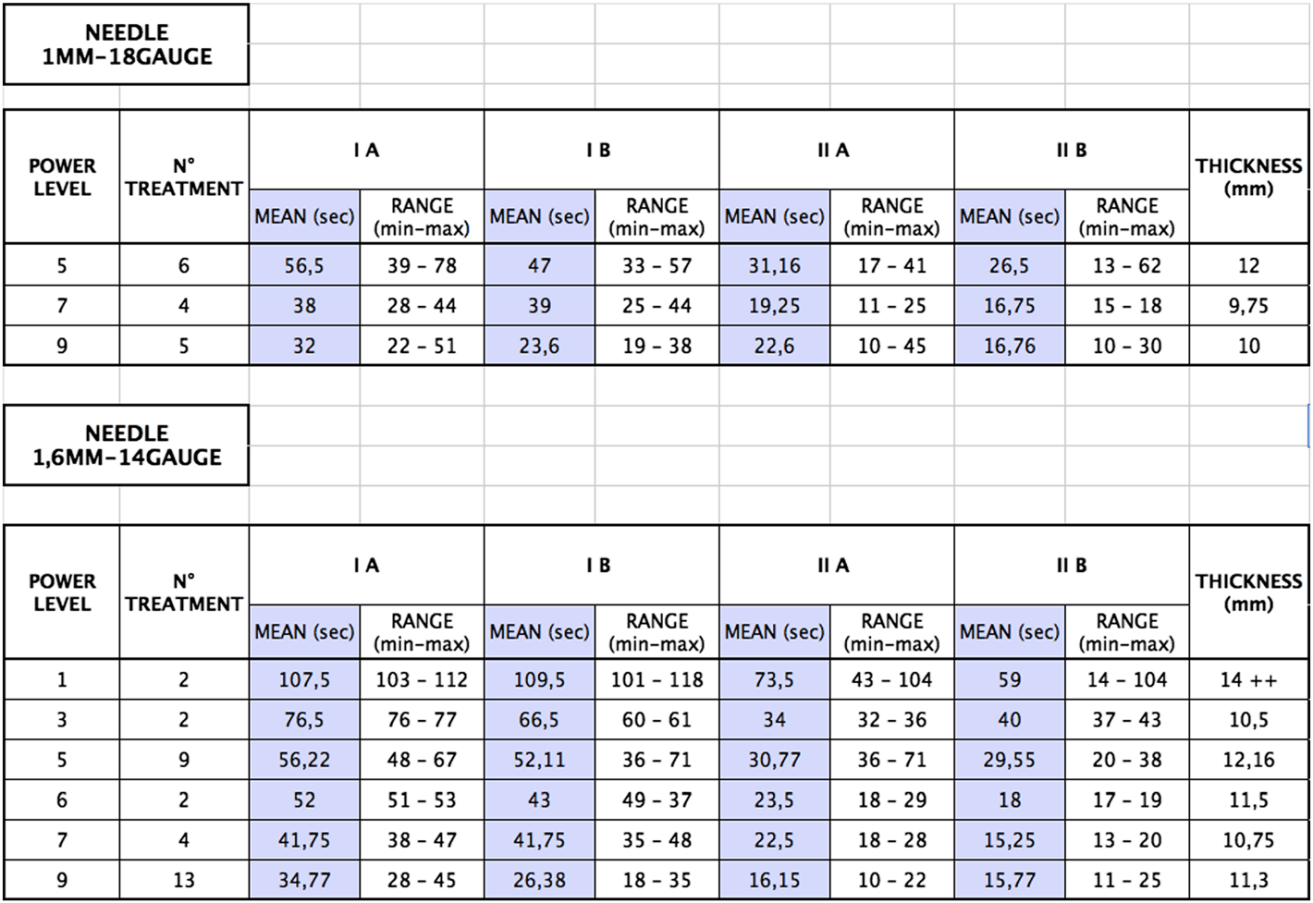
Relation between power level, needle’s caliper, coagulation time and thickness.

The statistical analysis of the data relating to coagulation time arising from the combination of the two modalities of needles (1 mm and 1.5 mm) with the three modalities of current used (Power levels 5, 7 and 9) are shown in the following Figure 3.

**Figure 4:**
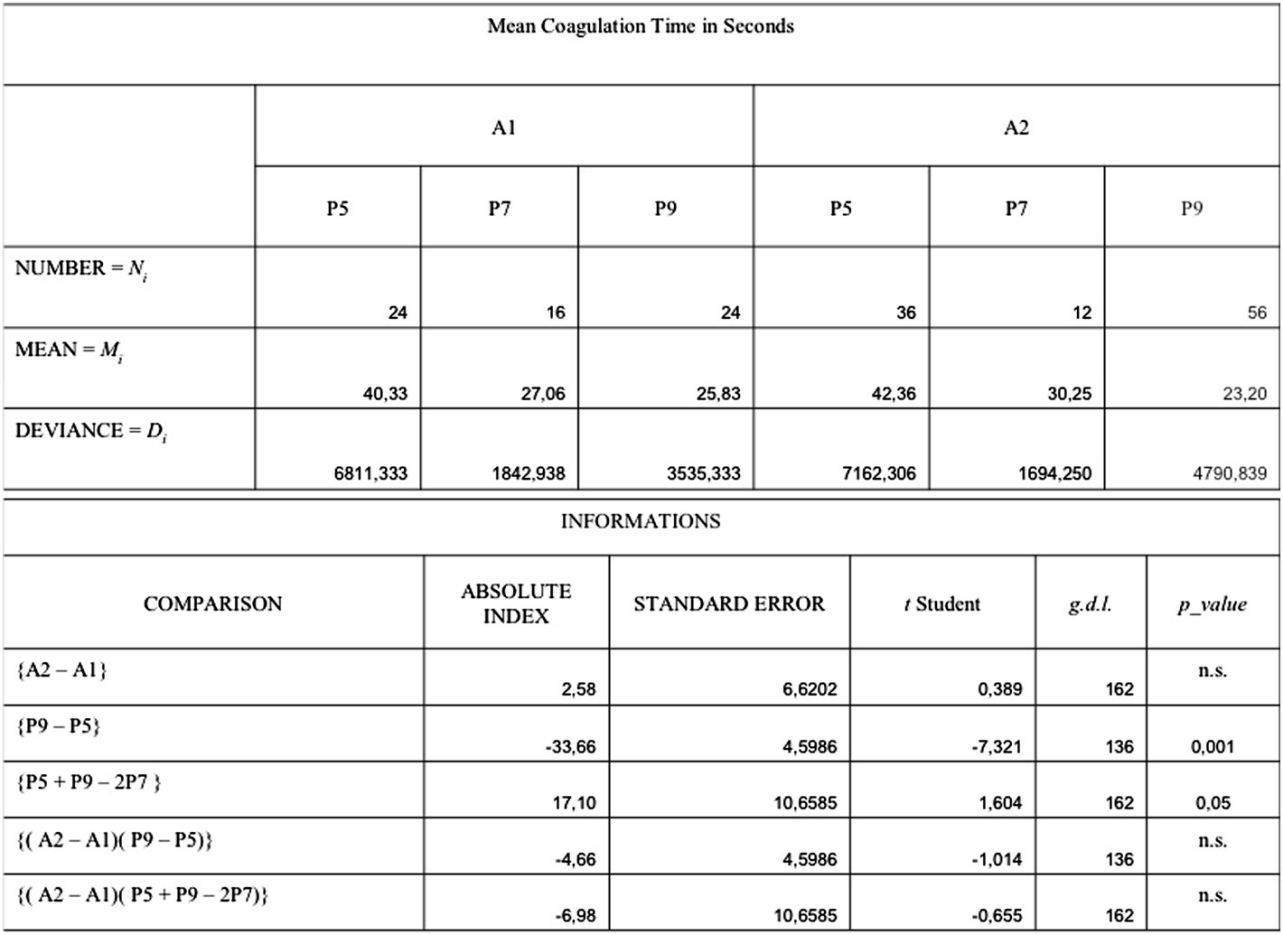
Statistical analysis. A1 = Needle: 1 mm; A2 = Needle: 1.5 mm; P5 = Power Level: 5; P7 = Power Level: 7; P9 = Power Level: 9.

The six combinations give rise to the five linearly independent comparisons shown in Figure 4.

The comparison between needles caliber (A2 – A1) and coagulation times highlight a shorter duration (2.58 sec.) with 1 mm needles compared with 1.5 mm with no statistical significance of the comparison.

However, the use of thin needles could cause incomplete coagulation in the area between them and, as a result of their flexibility, a deviation from the desired trajectory on the parenchyma.

The comparison between power level 9 and level 5 (P9 – P5), highlights that average coagulation times are dependent from power level and independent from the needle caliber.

There is a lower average coagulation time (33.66 seconds) with power level 9 respect to level 5. This comparison is statistically very significant (P value < 0.001).

The comparison between the various power levels 5, 7 and 9 (P5 + P9 – 2P7) showed a linearity of the effects of the different power levels on coagulation time (Coagulation time diminishes in a linear way, and systematically, with the increase in level of the power used).

The interaction {( A2 – A1)( P9 – P5)} shows that the lower coagulation time due to the higher level of power is less with the 1.5 mm needles than those of less size (1 mm), but does not show a statistical significance (P value > 0.05).

Lastly, the interaction {( A2 – A1)( P5 + P9 – 2P7)} shows the existence of a greater linearity in the power level effect on coagulation time with 1 mm needles than with those of 1.5 mm. Such interaction is not significant (P value >0.05).

## Discussion

Renal tumour surgery has evolved notably since the classical description of radical nephrectomy by Robson in 1963. As well as the laparoscopic approach, the most significant change has been the establishment of nephron sparing surgery as the gold standard for T1a renal neoplasms [13].

NSS brings results analogous to radical nephrectomy from the oncological point of view, with an increase in overall survival with respect to the morbidity and mortality correlated to the complete removal of the kidney [14].

The principal difficulty in NSS is the control of bleeding, which can be obtained with clamping of the renal vessels, through compression of the parenchyma, or with the use of several devices [15,16].

Various surgical instruments for tissue coagulation, haemostasis and parenchyma dissection are available along with sealants and haemostatic agents.

Instruments include the Water Jet Dissector, the Argon Beam Coagulator, the Tissuelink Floating Ball, the Harmonic Scalpel, Microwave Tissue Coagulation, Laser and Bipolar Coagulation.

Radiofrequency coagulation, born in the 90’s for ablation of liver neoplasms, and has been successively used to facilitate hepatic resection. The technique consisted of coagulating the liver parenchyma around the neoplastic nodule with a monopole “cooled-tip” needle to allow transection by scalpel. Later described in major liver resections [10], the technique is commonly known as “Radio-frequency assisted liver resection” (RFA-LR) [17].

In this connection, we planned and realized a generator and a dedicated multi-needle inline probe [11,12].

The “comb” arrangement of the electrode-needles permits the realization of “slices” of coagulation of the parenchyma around the neoplastic nodules, or along anatomic planes with closure of the blood and biliary vessels, without the Pringle manoeuvre and with little or no blood loss. The coagulated tissue can be transected by means of a scalpel.

That principle can be applied in theory to all parenchymas.

In literature there are data from several research groups on renal partial resection in animal models.

We mention Ong [18], who uses a bipolar electro-cauterizing device consisting of 2 electrode needles connected to a standard generator. Each polar resection needed about 20 “passes” of the instrument along with considerable time.

The author also mentioned the need for an impedance monitoring system to determine the completion of coagulation of the renal parenchyma.

In 2005, Mahvi [19] used a probe consisting of “plate” electrodes of 1 x 5 mm placed 1.5 cm from each other, with the energy applied between each pair of electrodes in bipolar mode, one pair at a time, with an interval of 600 milliseconds. A specific algorithm based on the impedance reached between each pair of electrodes was used to control the power delivered.

Morris [20] realized a probe with 6 inline needles connected to a RITA 1500 generator (RITA Medical System, Mountain View, CA, USA) able to produce energy at 460 Hz, with maximum power of 150 W; the power level was selected by the operator according to the thickness of the parenchyma to be coagulated.

Habib [21] has developed a 4-needle probe with a rhomboidal arrangement, called Habib 4X.

This probe has been used in the clinical setting for liver resections, partial renal resections, and for distal pancreasectomies.

According to the authors [21], NSS through that instrument is feasible and with a safety factor comparable with standard techniques without compromising the oncological outcome; however, the cost of the probe is relatively high.

RFA is therefore evidently valid also in the field of the renal parenchyma.

However, emerging from the literature is the necessity of a dedicated generator with a specific work algorithm and of a multi-electrode probe to reduce operating times.

Our system uses a multi-electrode fixed-needle probe, without the need to position manually each electrode, from which the energy is distributed automatically in each parenchymal section independently of its impedance.

This method allows for greater use of the system also for future applications such as laparoscopic probes.

The algorithm for the automatic shutoff of the electrodes allows the operator to follow and terminate automatically the treatment in each needle-to-needle zone, avoiding charring and, at the same time, guaranteeing coagulation of the tissue.

This first part of the project has been directed at the realization of the dedicated generator and the identification of the fundamental functional parameters for the renal parenchyma. The results are useful preparation for the execution of experimentation on the in vivo animal model and the realization of a laparoscopic probe.

## Conclusion

Our experimentation on a bench model has shown that the apparatus, consisting of an automatic and original power unit, and of a 4-electrode inline probe is able to carry out effective and homogeneas coagulation of the resection “slice”.

The coagulated tissue allows application of stitches on the parenchyma and the calyceal system.

Coagulation times are short; the generator delivers energy automatically thanks to the automatic impedance checking with switching off when optimal coagulation is reached.

The best caliber for the needle-electrodes turned out to be 1.5 mm as offering sufficient rigidity to maintain trajectory and parallel condition between them.

The appropriate power level is 5.

Lower power causing longer times and thicker “slices” of necrosis, while higher levels bring shorter coagulation times, but with the risk of causing charring, non-homogeneous coagulation and difficulty in removal of the probe.

The comb configuration of the probe allows adequate coagulation in a limited time. The coagulated tissue lends itself to division by means of scalpel.

The probe is easily used both during insertion and removal.

## Competing interests

The authors declare that they have no competing interests.

## Authors’ contributions

1- Study concept and design: PR; 2- Acquisition of data: ADM, ER; 3- Drafting of the manuscript: MM; 4- Critical revision of the manuscript for important intellectual content: PB; 5- Statistical analysis: CC; 6- Administrative, technical, and material support: MM, RB, LC; 7- Study supervision: GV; 8- Design of the generator and probe and acquisition of data relating to the functioning of the apparatus: PM, LI, VQ. All authors read and approved the final manuscript.

## Pre-publication history

The pre-publication history for this paper can be accessed here:

http://www.biomedcentral.com/1471-2490/14/7/prepub

## References

[B1] Jayson M Sanders H Incrased incidence of serendipitously discovered renal cell carcinoma Urology 1998 51 203 10.1016/S0090-4295(97)00506-2 9495698

[B2] Kutikov A Fossett LK Ramchandani P Tomaszewski JE Siegelman ES Banner MP Van Arsdalen KN Wein AJ Malkowicz SB Incidence of benign pathologic findings at partial nephrectomy for solitary renal mass presumed to be renal cell carcinoma on preoperative imaging Urology 2006 68 4 737 740 10.1016/j.urology.2006.04.011 17070344

[B3] Kane CJ Mallin K Ritchey J Cooperberg MR Carroll PR Renal cell cancer stage migration: analysis of the National Cancer Data Base Cancer 2008 113 1 78 83 10.1002/cncr.23518 18491376

[B4] Fergany AF Hafez KS Novick AC Long-term results of nephron sparing surgery for localized renal cell carcinoma: 10-year followup J Urol 2000 163 2 442 445 10.1016/S0022-5347(05)67896-2 10647650

[B5] McKiernan J Simmons R Katz J Russo P Natural history of chronic renal insufficiency after partial and radical nephrectomy Urology 2002 59 6 816 820 10.1016/S0090-4295(02)01501-7 12031359

[B6] Lesage K Joniau S Fransis K Van Poppel H Comparison between open partial and radical nephrectomy for renal tumors: perioperative outcome and health related quality of life Eur Urol 2007 51 3 614 620 10.1016/j.eururo.2006.10.040 17097216

[B7] Van Poppel H Da Pozzo L Albrecht W Matveev V Bono A Borkowski A Colombel M Klotz L Skinner E Keane T Marreaud S Collette S Sylvester R A prospective, randomised EORTC intergroup phase 3 study compating the oncologic outcome of elective nephron sparing surgery and radical nephrectomy for low stage renal cell carcinoma Eur Urol 2011 59 4 543 552 10.1016/j.eururo.2010.12.013 21186077

[B8] Gupta GN Peterson J Thakore KN Pinto PA Linehan WM Bratslavsky G Oncological outcomes of partial nephrectomy for multifocal renal cell caricnoma greater than 4 cm J Urol 2010 184 1 59 63 10.1016/j.juro.2010.03.035 20478582 PMC3197267

[B9] Galanakis I Vasdev N Soomro N A review of current hemostatic agents and tissue sealants used in laparoscopic partial nephrectomy Rev Urol 2011 13 3 131 138 22110396 PMC3221553

[B10] Navarra G Lorenzini C Currò G Basaglia E Habib NH Early result after radiofrequency-assisted liver resection Tumori 2004 90 1 32 35 15143968 10.1177/030089160409000108

[B11] Anselmo A Rossi P De Mayo A Manzia TM Iaria G Toti L Tisone G Radiofrequency-assisted right hemihepatectomy by using a new incremental bipolar generator combined with liver hanging maneuver Minerva Chir 2011 66 5 495 499 22117214

[B12] Rossi P De Majo A Mattei M Gaspari AL La resezione epatica mediante radiofrequenza bipolare: studi sperimentali su fegato di maiale I supplementi di Tumori 2004 3 5 S56 S58

[B13] Robson CJ Radical nephrectomy for renal cell carcinoma J Urol 1963 89 37 42 13974490 10.1016/S0022-5347(17)64494-X

[B14] Marszalek M Meixl H Polajnar M Rauchenwald M Jeschke K Madersbacher S Laparoscopic and open partial nephrectomy: a matched-pair comparison of 200 patients Eur Urol 2009 55 5 1171 1178 10.1016/j.eururo.2009.01.042 19232819

[B15] Stifelman MD Sosa RE Nakada SY Shichman SJ Hand Assisted laparoscopic partial nephrectomy J Endourol 2001 15 161 164 10.1089/089277901750134467 11325086

[B16] Ramakumar S Roberts WW Fugita OE Colegrove P Nicol TM Jarrett TW Kavoussi LR Slepian MJ Local hemostasis during laparoscopic partial nephrectomy using biodegradable hydrogels: initial porcine results J Endourol 2002 16 489 494 10.1089/089277902760367458 12396442

[B17] Weber JC Navarra G Jiao LR Nicholls JP Jensen SL Habib NA New technique of liver resection using heat coagulative necrosis Ann Surg 2002 236 5 560 563 10.1097/00000658-200211000-00004 12409660 PMC1422612

[B18] Ong AM Bhayani SB Hsu TH Pinto PA Rha KH Thomas M Nicol T Su LM Bipolar needle electrocautery for laparoscopic partial nephrectomy without renal vascular occlusion in a porcine model Urology 2003 62 6 1144 1148 10.1016/S0090-4295(03)00689-7 14665379

[B19] Haemmerich D Schutt DJ Will JA Striegel RM Webster JG Mahvi DM A device for radiofrequency assisted hepatic resection Conf Proc IEEE Eng Med Biol Soc 2004 4 2503 2506 17270781 10.1109/IEMBS.2004.1403721

[B20] Haghighi KS Steinke K Hazratwala K Kam PC Daniel S Morris DL Controlled study of Inline radiofrequency coagulation-assisted partial nephrectomy in sheep J Surg Res 2006 133 2 215 218 Epub 2006 Feb 7 10.1016/j.jss.2005.12.026 16464470

[B21] Pai M Spalding D Jiao L Habib N Use of bipolar radiofrequency in parenchymal transection of the liver, pancreas and kidney Dig Surg 2012 29 43 47 10.1159/000335732 22441619

